# Clinical Experience with Scleral Lens Wear After Corneal Cross-Linking in Keratoconus: Longitudinal Keratometric Outcomes

**DOI:** 10.3390/jcm15072764

**Published:** 2026-04-06

**Authors:** Yoo Young Jeon, Nahyun Park, Yea Eun Lee, Jeewon Han, Chung Min Lee, Carson Yu, Alison Hong, Kyu Sang Eah, Ho Seok Chung, Jae Yong Kim, Hun Lee

**Affiliations:** 1 Department of Ophthalmology, Asan Medical Center, College of Medicine, University of Ulsan, Seoul 05505, Republic of Korea; cheese_sauce@naver.com (Y.Y.J.); laurenpark66@gmail.com (N.P.); yeaeun812@gmail.com (Y.E.L.); jenny4132@naver.com (J.H.); chungminlee1215@gmail.com (C.M.L.); cyu993@gmail.com (C.Y.); kseah0124@gmail.com (K.S.E.); chunghoseok@gmail.com (H.S.C.); jykim2311@amc.seoul.kr (J.Y.K.); 2 Department of Ophthalmology, Chungnam National University Hospital, Daejeon 35015, Republic of Korea; 3 Department of Ophthalmology, School of Medicine and Health Sciences, The George Washington University, Washington, DC 20052, USA; ahong@mfa.gwu.edu; 4 Department of Ophthalmology, Brain Korea 21 Project, College of Medicine, University of Ulsan, Seoul 05505, Republic of Korea; 5 Center for Cell Therapy, Asan Medical Center, Seoul 05505, Republic of Korea

**Keywords:** keratoconus, corneal cross-linking, scleral lens, corneal astigmatism

## Abstract

**Objectives:** To investigate the effectiveness of scleral lenses following corneal cross-linking (CXL) in patients with progressive keratoconus. **Methods:** This retrospective study analyzed patients with keratoconus who underwent CXL and subsequently used scleral lenses for at least one year. A total of 21 patients (23 eyes) with progressive keratoconus were included. CXL was performed using the epithelium-off cross-linking technique following the Dresden protocol. Corneal astigmatism (CA), white-to-white distance, thinnest corneal thickness, and central corneal thickness (CCT) were assessed using corneal topography and tomography. Data were collected before CXL, at one month postoperatively, at the time of scleral lens prescription, and at 1, 3, 6, and 12 months after scleral lens use. **Results:** The mean age of the patients was 24.6 ± 4.6 years. The baseline uncorrected visual acuity (logMAR) before CXL was 1.05 ± 0.52. The baseline Kmax and CA were 53.01 ± 6.99 and −5.83 ± 3.23 D, respectively. The mean CCT was 444.09 ± 61.82 µm. The mean interval between CXL and scleral lens prescription was 6.75 ± 8.49 months. Following CXL, the Kmean decreased from 49.61 to 45.79 (*p* = 0.056), Kmax decreased from 53.01 to 51.32 (*p* = 0.053), and CA decreased from −5.83 to −4.91 D (*p* = 0.051), albeit statistically non-significant. At the 12-month follow-up after scleral lens prescription, Kmean and Kmax values remained stable, indicating no disease progression. Compared with baseline values before CXL, Kmean, Kmax, and CA were significantly reduced at the 12-month follow-up after scleral lens prescription (*p* = 0.047, *p* = 0.049, and *p* = 0.042, respectively). Scleral lens-corrected visual acuity was significantly better than habitual corrected visual acuity at all follow-up time points. **Conclusions:** Scleral lens application following CXL was associated with improved corrected visual acuity, and corneal keratometric values remained stable during lens wear.

## 1. Introduction

Keratoconus is a non-inflammatory, asymmetrical, progressive ectatic condition characterized by corneal thinning and protrusion into a conical shape. It typically develops during adolescence and progresses until the third or fourth decade of life, leading to corneal astigmatism (CA) and reduced vision [1,2]. As these patients are relatively young, the resulting visual impairment significantly affects their vision-related quality of life, limiting their ability to read and move while also causing emotional distress [3]. In the early stages of keratoconus, spectacles can be used to manage visual impairment. However, as the disease advances, rigid gas permeable (RGP) or scleral lenses become necessary. These lenses create a tear film between the lens and the cornea, compensating for corneal irregular astigmatism. If keratoconus progresses to a stage where these lenses are insufficient, surgical intervention is required. Corneal cross-linking (CXL) is the most commonly performed procedure for mild to moderate keratoconus, whereas corneal transplantation may be necessary in advanced cases [4]. 

CXL is a procedure that strengthens the cornea by applying riboflavin (vitamin B2) followed by ultraviolet A (UVA; 370 nm) irradiation, inducing a photochemical reaction that enhances covalent bonding in corneal collagen tissue [5]. The epithelium-off CXL technique, also known as the Dresden protocol, was introduced by Spoerl et al. [6]. This method uses 0.1% riboflavin in 20% dextran combined with UVA light exposure (365–370 nm) [6]. Since its introduction, numerous studies have reported the safety and efficacy of CXL with riboflavin and UVA [7,8,9]. In the first randomized controlled trial (RCT) on CXL, 22 eyes with progressive keratoconus and Kmax values between 48 and 72 D were treated. Keratoconus progression was halted in all eyes, with 16 (70%) demonstrating regression and a mean Kmax reduction of 2.01 D [10]. A subsequent study comparing 48 control eyes that did not undergo CXL with 46 eyes that did found a significant Kmax reduction in the CXL-treated group at the 36-month follow-up [5].

Scleral lenses are large-diameter, rigid, gas-permeable lenses that correct astigmatism by maintaining a tear fluid layer between the lens and the cornea [11,12]. Consequently, these lenses are widely used to manage astigmatism in patients with corneal ectasia, keratoconus, residual astigmatism following corneal transplantation, irregular astigmatism, and other ocular surface diseases or corneal dystrophies [13,14]. A previous study demonstrated the therapeutic efficacy of scleral lenses in 121 cases of keratoconus, 29 cases of post-penetrating keratoplasty, 31 cases of other ocular surface diseases, and 28 cases of irregular astigmatism [15]. The mean decimal best-corrected visual acuity was 0.8, indicating a substantial improvement in visual acuity [15]. In cases of keratoconus, patients using scleral lenses achieve better best-corrected visual acuity than those using glasses or RGP lenses, and the safety of scleral lenses has been confirmed over a follow-up period exceeding one year [16,17]. However, no definitive evidence suggests that scleral lens use prevents keratoconus progression, as 23 eyes (14.6%) of 157 patients with keratoconus experienced a decline in corrected visual acuity due to disease progression within a few years of scleral lens use [16].

Given the need for long-term treatment strategies that not only suppress keratoconus progression but also provide visual correction, this study aimed to investigate the effectiveness of scleral lenses following CXL in managing keratoconus and preventing disease progression.

## 2. Materials and Methods

This retrospective study analyzed the medical records of patients diagnosed with keratoconus between 2019 and 2024 at Asan Medical Center in Seoul, Republic of Korea. Patients who underwent CXL and were subsequently prescribed scleral lenses for at least one year were included. All procedures conformed to the Declaration of Helsinki, and the study was approved by the institutional review board of the Asan Medical Center.

A total of 21 patients (23 eyes) were enrolled, all of whom were diagnosed with keratoconus based on the Amsler–Krumeich classification, corneal topography, corneal tomography, and slit lamp examination. Progressive keratoconus was defined as keratoconus with any of the following: (1) an increase in astigmatism of ≥1 diopter (D); (2) significant changes in the axes; (3) an increase in maximum keratometry (Kmax) of ≥1 D; or (4) a decrease in corneal thickness of ≥25 µm [18].

**Figure 1:**
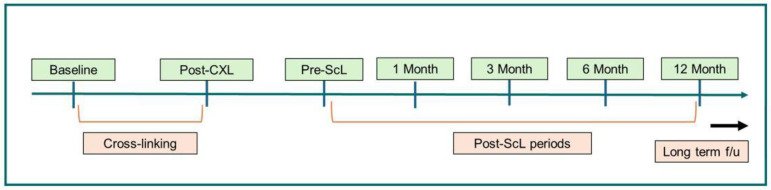
Timeline of the retrospective study.

Data were collected before CXL; at one month postoperatively; at the time of scleral lens prescription; and at 1, 3, 6, and 12 months after scleral lens treatment. The timeline of the analyzed time points is illustrated in Figure 1.

Mean keratometry (Kmean), maximum keratometry (Kmax), corneal astigmatism (CA), and white-to-white distance were measured with ORBSCAN (Bausch & Lomb, Rochester, NY, USA), while thinnest corneal thickness (TCT) and central corneal thickness (CCT) were measured with CASIA2 (Tomey, Nagoya, Japan). Measurements were performed using the same device for each parameter across all subjects to minimize inter-device variability. At each visit, uncorrected visual acuity (UCVA), habitual corrected visual acuity (HCVA), scleral lens-corrected visual acuity (SLCVA), intraocular pressure, manifest refraction, and auto-refraction were assessed.

The exclusion criteria were as follows: patients with corneal diseases or corneal lesions other than keratoconus; patients with a history of other ocular diseases, including glaucoma or retinal disorders; patients with a history of ocular trauma or ocular surgery; and those who discontinued lens wear within the first year. Patients included in the study had no other diagnosed systemic diseases.

CXL was performed using epithelium-off cross-linking according to the Dresden protocol. First, an 8 mm diameter area of the epithelium was removed, and 0.1% riboflavin was instilled every 180 s for 30 min. Ultraviolet (UV) light at 365 nm was then applied at a power of 9 mW/cm^2^ for 13 min 20 s, delivering a total energy of 7.2 J/cm^2^ [19]. Therapeutic lenses were applied postoperatively. The postoperative eye drops included 0.5% levofloxacin (Cravit^®^ ophthalmic solution 0.5%; Santen Pharmaceutical Co., Ltd., Osaka, Japan) and 0.1% fluorometholone (Fumelone eye drops 0.6 mL; Hanlim Pharm. Co., Seoul, Republic of Korea), both administered four times daily. Additionally, 0.5% carboxymethylcellulose (Refresh Plus 0.4 mL; Allergan, Marlow, UK) was used four times daily.

The scleral lens used in this study was the Onefit lens, which is fabricated from a gas-permeable fluorosilicone acrylate polymer (Acuity 100, Acuity Polymers Inc., New York, USA) and has an oxygen permeability (Dk) of 100 × 10^−11^ cm^2^ · mL O_2_/s · mL · mmHg. The lens has an overall diameter ranging from 14.1 to 15.3 mm, with a standard size of 14.9 mm. The lenses were fitted by adjusting the sagittal height according to the manufacturer’s fitting guide. Corneal clearance was evaluated using a slit lamp at a 40° oblique position. The ideal clearance after lens insertion was 200–225 μm at the point of highest corneal elevation.

### Statistical Analysis

All statistical analyses were performed using SPSS for Windows (version 25.0; SPSS Inc., Chicago, IL, USA). The Shapiro–Wilk test was used to assess data normality. The efficacy of CXL and scleral lens prescription was analyzed using a paired *t*-test. A *p*-value < 0.05 was considered statistically significant.

## 3. Results

Twenty-one patients (23 eyes) were included, with a mean age of 24.6 ± 4.6 years. Men accounted for 87% of the total population. The baseline UCVA (logMAR) before CXL was 1.05 ± 0.52, and the mean spherical equivalent was −5.17 ± 5.39 D (Table 1). The Kmax measured using corneal topography was 53.01 ± 6.99 D, and the CA was −5.79 ± 3.34 D. The mean CCT was 450.73 ± 57.78 µm. The mean interval between CXL and scleral lens prescription was 6.75 ± 8.49 months, ranging from 1 to 33 months. All patients had endothelial cell density within the normal range.

Table 2 shows the measurements obtained before CXL, 1 month post-CXL, at the time of scleral lens prescription (pre-ScL), and at 1, 3, 6, and 12 months after scleral lens wear. Keratometric values measured at 1 month post-CXL demonstrated an apparent flattening trend; however, these early postoperative measurements were not considered representative of stable corneal biomechanics because transient epithelial remodeling, edema, or haze may influence topographic readings during the immediate postoperative period. Therefore, the pre-ScL time point was regarded as the stable 1-month post-CXL baseline for longitudinal evaluation. From pre-ScL to 12 months after scleral lens wear, Kmean, Kmax, and CA did not differ significantly (all *p* > 0.05), indicating maintenance of corneal stability during lens wear.

Compared with baseline (pre-CXL), Kmean, Kmax, and CA at 12 months after scleral lens prescription were significantly lower (*p* = 0.047, *p* = 0.049, and *p* = 0.042, respectively), reflecting the overall clinical course following combined CXL and scleral lens management. CCT and TCT decreased after CXL, increased at 1 month after scleral lens wear (*p* < 0.05), and then remained stable throughout follow-up.

Regarding visual outcomes, UCVA showed numerical improvement at 12 months, but the change was not statistically significant (*p* = 0.068). SLCVA was consistently better than HCVA at all time points, with no significant longitudinal differences after lens prescription.

## 4. Discussion

We aimed to investigate the clinical effect of prescribing scleral lenses after CXL in patients with keratoconus. Previous studies have reported the efficacy and safety of CXL in patients with progressive keratoconus, while other studies have demonstrated the effectiveness of scleral lenses in patients with relatively stable keratoconus [5,10,15,16,17]. However, the clinical effect of combining these two treatment options at specific intervals to stabilize the progression of keratoconus has not been investigated.

The Kmean, Kmax, and CA values significantly decreased after CXL compared to preoperative values, suggesting corneal flattening consistent with previous studies [10,20,21,22]. However, by the time scleral lenses were prescribed, patients showed an increase of 3.33 D in Kmean and 0.6 D in Kmax, indicating variability in keratometric measurements, although these changes were not statistically significant. After scleral lens prescription, Kmean and Kmax remained stable, with no statistically significant differences observed between the pre-scleral lens and 12-month follow-up results. Several previous studies have reported that keratometric values after CXL may change over time [23,24]. There are no clearly defined clinical criteria for keratoconus re-progression; however, in a systematic review, 25 studies defined a change in Kmax of ≥1.0 D after treatment as indicative of re-progression [25]. In another study involving 131 eyes of patients with keratoconus who underwent CXL, those with a postoperative increase in Kmax >2 D were considered non-responders to CXL. Risk factors for non-responsiveness included younger age, high astigmatism (>4.3 D), thin corneas (<480 µm), poor initial visual acuity (CDVA ≥ 0.3 logMAR), and atopic dermatitis [26]. Although CXL is widely used to reduce the risk of keratometric worsening, long-term corneal measurements may vary among individuals, suggesting that continued monitoring and appropriate visual rehabilitation are important.

Visual acuity tended to improve over the 12 months following scleral lens wear compared to baseline. Additionally, SCLVA was higher at all time points compared to HCVA at baseline, post-CXL, and pre-scleral lens insertion. These findings suggest that keratometric measurements remained stable and that visual acuity was effectively improved with scleral lens wear over the 12-month period.

Previous studies have similarly reported improvements in corrected visual acuity at long-term follow-up after either CXL or scleral lens prescription [5,13,27]. In a study by Ferdi et al. involving 976 eyes, mean CDVA improved from baseline by 3.7 logMAR letters (*p* < 0.001) at 1 year and 6.9 letters (*p* < 0.001) at 5 years [28]. However, other studies have shown that a subset of eyes may still demonstrate late keratometric steepening or continued changes after treatment. Koller et al. reported that approximately 7–11% of eyes were classified as non-responders, with ongoing increases in keratometric values after CXL [29]. The U.S. multicenter trial by Hersh et al. similarly noted that some eyes exhibited postoperative increases in Kmax despite treatment [30]. These findings suggest that, while CXL is effective for most patients, maintaining stable keratometry remains a clinical challenge in certain cases, highlighting the need for continued monitoring and additional strategies for long-term visual and structural management. Given the small sample size (n = 21), our study was not able to detect rare subgroups such as non-responders. Nevertheless, keratometric values remained stable during scleral lens wear, with no significant increases observed throughout follow-up.

In clinical practice, scleral lens wear is commonly associated with improved visual acuity, with keratometric values typically remaining stable in patients with keratoconus. Knoeri et al. compared the outcomes of corrective glasses, RGP lenses, and scleral lenses in patients with stage 4 keratoconus [31]. No significant differences in mean best-corrected visual acuity (BCVA, logMAR) were found between the scleral lens and RGP lens groups. However, mean BCVA values were significantly better with both scleral and RGP lenses than with corrective glasses. Additionally, changes in higher-order aberrations, including total higher-order aberrations, coma, and trefoil, were significantly lower with scleral lenses than with RGP lenses [31].

In another study, Ksteep, Kflat, and Kmax values were significantly lower immediately after scleral lens removal compared to 1 week after discontinuation of scleral lens wear [32]. Specifically, Ksteep was 0.7 D lower (*p* < 0.001), Kflat was 0.5 D lower (*p* = 0.037), and Kmax was 1.1 D lower (*p* < 0.001) immediately after lens removal. Although scleral lenses do not physically contact the cornea, corneal curvature appears to be influenced by lens wear during the wearing period in patients with keratoconus [30]. Therefore, the present findings demonstrate that CXL effectively halts keratoconus progression in patients with progressive disease, and subsequent scleral lens prescription improves visual acuity and stabilizes corneal curvature.

As keratoconus progresses and the cornea becomes increasingly conical, certain regions undergo thinning. Therefore, follow-up assessments of TCT and CCT are useful for monitoring disease progression. In this study, CCT decreased from baseline following CXL but increased significantly and recovered by the first month after scleral lens wear, remaining stable through 12 months. This suggests that scleral lens prescription after CXL does not contribute to corneal thinning. Greenstein et al. reported that CCT significantly decreased until 1 month after CXL but recovered at 3 to 6 months, with no significant difference from baseline values, which aligns with our findings [33,34]. Therefore, the observed CCT recovery in this study reflects the natural course of post-CXL corneal thickness changes, with no additional effect attributable to scleral lenses.

This study has several limitations. First, it is difficult to attribute changes in corneal thinning or disease progression to a single intervention, as a direct comparison with outcomes of CXL alone was not possible. Further prospective studies with appropriate control groups are warranted to better clarify the role of scleral lenses in post-CXL keratoconus management. Second, differences in baseline corneal parameters and visual acuity, which may reflect varying stages of keratoconus, could have influenced the outcomes. Further research comparing outcomes in patients with stage 3 and 4 keratoconus based on the Amsler–Krumeich classification would be valuable. Third, long-term follow-up was not conducted. Given that keratoconus can progress for approximately 20 years, continuous monitoring and management are essential. In addition, the interval between CXL and scleral lens prescription varied widely among patients owing to the retrospective study design and differences in clinical indications for lens fitting. Although the primary analyses were based on within-eye longitudinal comparisons after lens prescription, this heterogeneity may have introduced potential confounding and should be considered when interpreting the results. Regarding the statistical analysis, although paired t-tests were used for prespecified pairwise comparisons between time points, this approach may increase the risk of Type I error due to multiple testing. A repeated-measures ANOVA or a mixed-effects model could provide a more statistically robust framework for longitudinal analysis and should be considered in future studies.

## 5. Conclusions

In conclusion, scleral lens prescription following CXL was associated with improved corrected visual acuity, and corneal keratometric values remained stable during lens wear throughout follow-up. These findings suggest that scleral lens wear does not adversely affect corneal stability after CXL while providing effective visual rehabilitation.

## Figures and Tables

**Table 1:** Preoperative baseline characteristics of all patients.

Parameters	Mean ± SD (Range)	Parameters	Mean ± SD (Range)
Age (years)	24.56 ± 4.63 (17–33)	Topography	
Sex (male/female)	20 (87%)/3 (13.0%)	Kmean (D)	49.61 ± 6.19 (40.45–66.45)
CXL-ScL Interval (month)	6.75 ± 8.49 (1–33)	Kmax (D)	53.01 ± 6.99 (41.60–68.50)
UCVA (logMAR)	1.05 ± 0.52 (0.30–2.00)	CA (D)	−5.83 ± 3.23 (−14.70–−2.30)
HDVA (logMAR)	0.55 ± 0.51 (0.05–2.00)	Autokeratometry	
Spherical power (D)	−12.02 ± 3.48 (−19.25–−2.25)	Kmean (D)	53.79 ± 4.42 (41.00–61.00)
Cylindrical power (D)	5.98 ± 2.31 (1.75–9.50)	Kmax (D)	50.95 ± 3.72 (40.12–57.00)
Spherical equivalent (D)	−9.02 ± 3.28 (−17.00–−1.38)	CA (D)	−6.12 ± 2.07 (−9.25–−1.75)
IOP (mmHg)	11.47 ± 1.85 (10–17)	TCT (µm)	417.88 ± 63.67 (289–486)
WTW (mm)	11.56 ± 0.42 (10.9–12.7)	CCT (µm)	444.09 ± 61.82 (330–515)
		ECD (cells/mm^2^)	2632.6 ± 337.4 (2075–3097)

Results are shown in Mean ± standard deviation and range. UCVA, uncorrected visual acuity; HDVA, habitual corrected visual acuity; IOP, intraocular pressure; D, diopters; CA, corneal Astigmatism; CXL, corneal cross-linking; Kmean, mean keratometry; Kmax, maximum keratometry; WTW, white-to-white; TCT, thinnest corneal thickness; CCT, central corneal thickness; CXL-ScL interval, time interval between crosslinking and scleral lens wear.

**Table 2:** Comparison of preoperative and postoperative parameters for corneal cross-linking.

Parameters	Baseline	Post-CXL	*p*-Value(a) *	Pre-ScL	1 Month	3 Month	6 Month	12 Month	*p*-Value(b) *	*p*-Value(c) *
UCVA (logMAR)	1.05	0.96	0.061	0.99	0.96	0.96	0.95	0.94	0.704	0.068
HCVA (logMAR) †	0.55	0.31	0.234	0.27					0.047	0.024
SLCVA (logMAR) §					0.20	0.19	0.17	0.22		
Topography										
Kmean (D)	49.61	45.79	0.056	49.12	50.14	50.34	49.82	49.22	0.893	0.047
Kmax (D)	53.01	51.32	0.053	51.92	53.14	53.41	52.73	52.02	0.455	0.049
CA (D)	−5.83	−4.91	0.051	−5.25	−5.61	−5.76	−5.40	−5.25	0.994	0.042
Autokeratometry										
Kmean (D)	−12.02	−13.28	0.608	−12.84	−12.25	−12.20	−11.50	−11.73	0.325	0.046
Kmax (D)	5.98	6.14	0.632	5.83	5.63	5.59	5.21	5.55	0.498	0.053
CA (D)	−9.02	−10.37	0.869	−9.92	−9.44	−9.41	−8.89	−8.96	0.680	0.533
TCT (µm)	417.88	387.22	0.050	398.66	415.13	417.13	406.19	414.50	0.170	0.724
CCT (µm)	444.09	412.72	0.123	423.97	458.50	463.84	452.72	447.75	0.576	0.666

Results are shown in Mean ± standard deviation and range. UCVA, uncorrected visual acuity; HDVA, habitual corrected visual acuity; IOP, intraocular pressure; D, diopters; CA, corneal Astigmatism; CXL, corneal cross-linking; ScL, scleral lens; Kmean, mean keratometry; Kmax, maximum keratometry; WTW, white-to-white; TCT, thinnest corneal thickness; CCT, central corneal thickness. * *p*-value(a) = *p*-value between baseline and Post-CXL, *p*-value(b) = *p*-value between Pre-ScL and 12 months after scleral lens prescription, *p*-value(c) = *p*-value between baseline and 12 months after scleral lens prescription. † *p*-values (HCVA) between baseline vs. Post-CXL, baseline vs. Pre-ScL were 0.234, 0.152, respectively. § *p*-values (SLCVA) between 1 Mo vs. 3 Mo, 3 Mo vs. 6 Mo, 6 Mo vs. 12 Mo, and 1 Mo vs. 12 Mo were 0.985, 0.341, 0.592, and 0.746, respectively.

## Data Availability

The datasets generated and/or analyzed during the current study are not publicly available due to patient privacy concerns and restrictions imposed by the Institutional Review Board. Participants did not provide consent for public sharing of their clinical data. However, the data may be available from the corresponding author on reasonable requests and with appropriate ethical approvals.
